# Skin infection caused by a novel strain of *Staphylococcus pseudintermedius* in a Siberian husky dog owner

**DOI:** 10.1099/jmmcr.0.005087

**Published:** 2017-03-20

**Authors:** Andrew R Robb, Elizabeth D Wright, Adele M. E Foster, Robert Walker, Colin Malone

**Affiliations:** ^1^​ Scottish Microbiology Reference Laboratories, Glasgow Royal Infirmary, New Lister Building, 10–16 Alexandra Parade, Glasgow G31 2ER, UK; ^2^​ Department of Microbiology, Dumfries and Galloway Royal Infirmary, Bankend Road, Dumfries DG1 4AP, UK; ^3^​ Department of Dermatology, Dumfries and Galloway Royal Infirmary, Bankend Road, Dumfries DG1 4AP, UK

**Keywords:** zoonosis, *Staphylococcus pseudintermedius*, Siberian husky, ecthyma-like, flucloxacillin

## Abstract

**Introduction.** *Staphylococcus pseudintermedius*, an opportunistic pathogen of dogs and cats, is rarely reported to cause infection in humans. Here, we describe a case of severe skin infection caused by *S. pseudintermedius*, in a 47-year-old male, a dog owner; to the best of our knowledge, this is the first such case reported from Scotland.

**Case presentation.** The patient presented with a short history of a severe ecthyma-like lesion on his forehead, with smaller lesions on his abdomen and legs. Bacterial culture revealed *Clostridium perfringens*, thought to be colonizing the wound, and a *Staphylococcus* species, identified as *S. pseudintermedius* by matrix-assisted laser desorption/ionization-time of flight MS and confirmed by molecular methods using a PCR-RFLP approach. The patient was treated with flucloxacillin, penicillin V and Fucibet cream, and recovered fully. Zoonotic infection was considered likely; however, screening swabs from his dogs grew *S. pseudintermedius* of a different clonal type. Both patient and dog strains carried *Staphylococcus intermedius* exfoliative toxin and leucocidin I, closely related to Panton–Valentine leucocidin, possibly contributing to the severity of the infection. *S pseudintermedius*, although coagulase positive, is normally negative by rapid slide clumping and latex agglutination tests routinely used to identify *Staphylococcus aureus*. Hence, *S. pseudintermedius* may easily be misidentified as a coagulase-negative staphylococcus and considered insignificant.

**Conclusion.** This is, to the best of our knowledge, the first reported case of a human *S. pseudintermedius* infection in Scotland. Zoonotic transmission of *S. pseudintermedius* between pets and owners has been shown. However, in this case zoonosis could not be confirmed.

## Abbreviations

CNS, coagulase-negative staphylococcus; MALDI-TOF, matrix-assisted laser desorption/ionization-time of flight; PVL, Panton–Valentine leukocidin; ST, sequence type.

## Introduction

*Staphylococcus pseudintermedius* belongs to a group of three closely related staphylococcal species (*S. pseudintermedius*, *Staphylococcus intermedius* and *Staphylococcus delphini*) known as the *S. intermedius* group [1]. *S. pseudintermedius* is a member of the normal flora, colonizing up to 90 %, of dogs, and is also a major opportunistic pathogen responsible for a wide range of infections including pyoderma, otitis, wound and urinary-tract infections in dogs, cats and horses, but has only rarely been isolated from humans [2]. However, there have been an increasing number of case reports documenting serious invasive infections caused by *S. pseudintermedius* in humans, including infected dog bite wounds, bacteraemia, pneumonia, brain abscesses and septic arthritis, most of which have been related to dog exposure [3]. While *S. pseudintermedius* is a coagulase-positive organism, the characteristics of its coagulase differs from *Staphylococcus aureus* coagulase [4]. The majority of medical microbiology departments use rapid latex slide agglutination tests to screen for *S. aureus*. These tests differentiate *S. aureus* from other staphylococcal species by detecting clumping factor and cell bound protein A. As these two factors are rarely present in *S. pseudintermedius*, up to 90 % of isolates tested will give a negative result. Therefore, the true incidence of human infection is unknown, but is likely to be higher than that reported. This is due to the pathogen being frequently misidentified, either as a coagulase-negative staphylococcus (CNS) and regarded as a contaminant, or as *S. aureus* [5]. Since the implementation of the matrix-assisted laser desorption/ionization-time of flight (MALDI-TOF) MS (Bruker), rapid and accurate identification of *S. pseudintermedius* is now possible, whereas previously it may have been misidentified as *S. aureus* or discarded based on a negative latex agglutination test [6].

Similar to *S. aureus*, *S. pseudintermedius* produces numerous virulence factors including toxins, such as haemolysins, exfoliative toxins, coagulase, thermonuclease, clumping factor and protein A, enterotoxins and a leucotoxin I (Luk-I), which is very similar to Panton–Valentine leucocidin (PVL) from *S. aureus*. However, as many of these virulence factors have yet to be characterized in detail, knowledge of the pathogenesis of *S. pseudintermedius* is very limited [7]. This case report details the clinical features, diagnosis and the phenotypic and molecular characterization of *S. pseudintermedius* isolates from an infected human and his colonized dogs to determine whether zoonotic transmission had occurred.

## Case Report

**Fig. 1.**
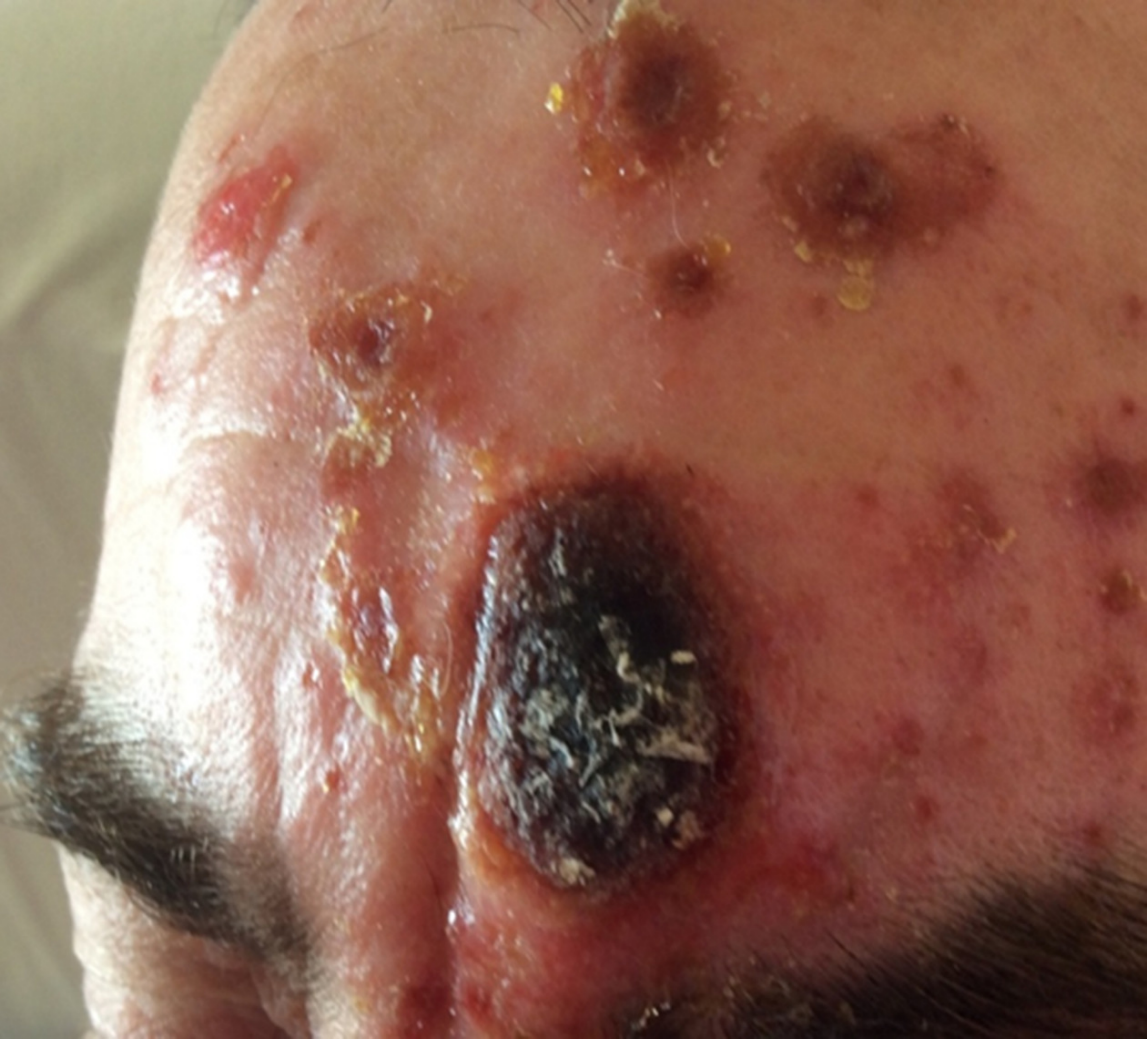
Appearance of the skin lesion at presentation.

A 47-year-old sawmill worker presented with a 1 week history of an ecthyma-like, painful, enlarging crusting lesion on his forehead, with several smaller satellite lesions (Fig. 1). Initially, a small red mark had appeared over the glabellar region of his forehead, which rapidly developed into the large eschar. There was no history of trauma to the area. He then developed very itchy satellite lesions on the forehead, abdomen and limbs. He had two reactive lymph nodes palpable in the left cervical chain, but was neither systemically unwell nor febrile. He reported a similar lesion on his forehead two years previously, which had responded rapidly to oral flucloxacillin. A swab from the main lesion grew *Clostridium perfringens* and a CNS identified by MALDI-TOF MS as *S. pseudintermedius*. No fungi were isolated from the lesion. The patient’s anterior nares were screened for colonization with *S. pseudintermedius*, which were negative.

The isolation of *C. perfringens* from the lesion was concerning and its significance uncertain. There were no features of clostridial soft tissue infection, no loss of tissue viability around the lesion and no evidence of skin necrosis, no bullae, blisters, bruising nor numbness, and no systemic toxicity. It was concluded that this organism was colonizing the lesion and not contributing to the infection.

**Fig. 2.**
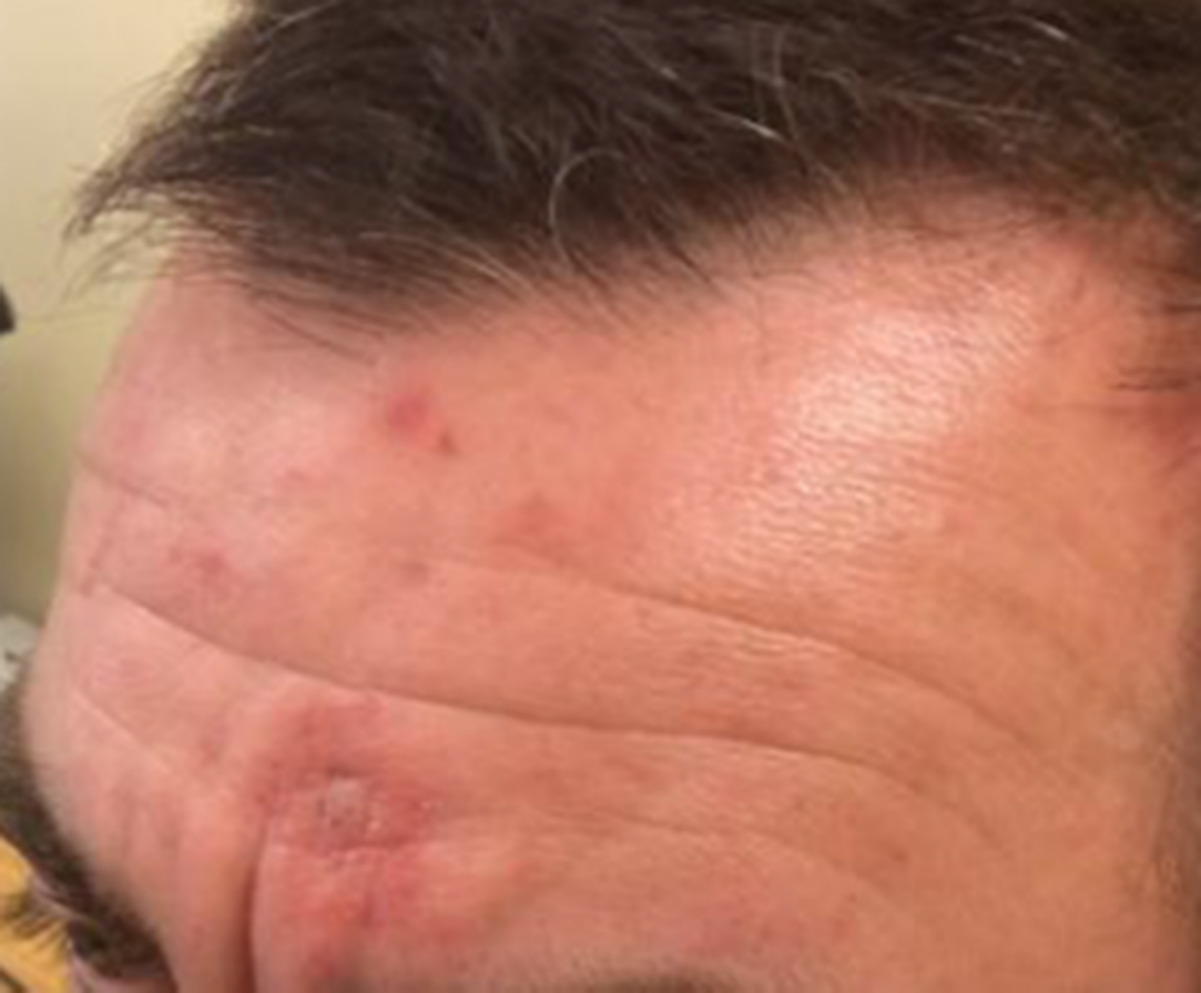
Appearance at 5 weeks post-treatment.

The patient was treated with oral penicillin V, 500 mg, and flucloxacillin, 500 mg, four times daily for 2 weeks. When these antibiotics were started, the *S. pseudintermedius* had not been fully identified and the significance of the *C. perfringens* was yet to be determined. It was, therefore, felt reasonable to include penicillin V with flucloxacillin. Fucibet cream was included to reduce inflammation, irritation and wound discharge. This was applied topically twice daily for several weeks, in conjunction with warm bathing with water/white vinegar twice daily until the eschar separated. The patient was given oral chlorphenamine, 4 mg, as required, for the itching. Five weeks later, his skin lesion had almost completely resolved (Fig. 2) with some residual areas of post-inflammatory hyperpigmentation. He was given a rescue course of oral flucloxacillin, 500 mg, four times daily, to use in the event of a further infection.

Three pet Siberian husky dogs resided with the male owner. The dogs were healthy, well cared for and had no skin lesions nor infection. The patient had no other close contact with dogs. Other members of the family had no current or past skin problems or infections.

## Investigations

Admission blood tests revealed a white cell count of 12.8×10^9^ cells l^−1^, CRP <3 mgl^−1^, and normal urea and electrolytes, liver function tests, glucose, IgG, IgA and IgM immunoglobulins, and a negative human immunodeficiency virus antibody test. The patient's wound swabs were cultured on Columbia blood and Sabouraud agar incubated in air and neomycin blood agar incubated anaerobically, for 48 h.

Each dog was screened for *S. pseudintermedius* colonization. Swabs were taken from the nostril, mouth, ear, forelimb and hind limb axillae, between the toes, and the anal margin. Swabs taken from the dogs were positive for *S. pseudintermedius*, identified by MALDI-TOF MS. Species identification was confirmed by PCR and PCR-RFLP as described elsewhere [8, 9].

PFGE and multi-locus sequence typing (MLST) was performed as described elsewhere [10, 11]. The PFGE analysis, using Bionumerics software version 7.6.1 (Applied Mathematics), with a similarity cut off of 80 % showed that the patient and dog isolates were unrelated (data not shown). This was confirmed by MLST, where the patient and dog isolates belonged to novel unrelated sequence types (STs) (ST673 allelic profile 4-72-3-1-8-4-2 and ST686 allelic profile 5-7-2-23-8-1-1, respectively).

Genes encoding toxins PVL, toxic shock syndrome toxin 1 and exfoliative toxins a, b were not detected by PCR [12]. However, both the patient and dog strains carried *S. intermedius* exfoliative toxin (SIET) and leucocidin I (*lukF/S*-PV), which is closely related to PVL [13, 14]. Antibiotic-susceptibility testing performed with the Vitek 2 system revealed the patient’s strain to be resistant to penicillin, confirmed by the presence of the *blaZ* gene [15], and susceptible to oxacillin and fusidic acid. The canine strains were susceptible to penicillin, confirmed by the absence of the *blaZ* gene, oxacillin and fusidic acid.

## Outcome and follow-up

The patient completed his treatment and recovered fully.

## Discussion

There have been relatively few studies on the rate of *S. pseudintermedius* colonization in humans and in those that have been performed low levels of colonization have been reported. In a study of humans and their pet dogs in Ontario, Canada, *S. pseudintermedius* was isolated from 4.1 % of healthy subjects [16], and a study from Japan, investigating rates of staphylococci in the oral cavities of healthy adults, found members of the *S. intermedius* group in 8.9 % of subjects [17].

*S. pseudintermedius* is a coagulase-positive organism, which is normally negative by rapid slide clumping test and by commercial latex agglutination tests that detect clumping factor, protein A and/or surface antigens in *S. aureus*. As a consequence, the risk that *S. pseudintermedius* is misidentified as a CNS is high in human diagnostic laboratories, as these commercial tests are routinely used to screen for rapid discrimination of *S. aureus* and CNS [7].

Clinical details concerning the nature of an infection are, of course, very significant for determining optimal microbiological laboratory processing of specimens. In this case, clinical information on the request form was lacking, and had it not been for the presence of a potentially significant pathogen, *C. perfringens*, and the severity of the lesion, the unknown staphylococcus would not have been fully identified. Identification of this isolate as *S. pseudintermedius* prompted the history of dog contact to be elicited from the patient. The significance of the *C. perfringens* remains uncertain; polymicrobial cultures from humans and dogs infected with *S. pseudintermedius* are common [18, 19]. While it has been reported that the virulence of *S. pseudintermedius* infections in humans may differ to those of companion animals, where the organism is often the primary pathogen, this organism certainly has the potential to be virulent in the human host.

At present, the true incidence of *S. pseudintermedius* as a human zoonotic pathogen is unknown [20]. However, reports of zoonotic transmission of meticillin-susceptible and -resistant *S. pseudintermedius* between dogs and humans have been published [21]. Guardabassi *et al.* [22] have investigated the occurrence of *S. pseudintermedius* in infected dogs, their owners and subjects with no daily contact with dogs. In the study, the dog owners were significantly more likely to carry *S. pseudintermedius* than the control group and they often carried the same strain as their dog. In this case, the strain infecting the patient was different to that of his colonized dogs, indicating that zoonotic transmission, between the patient and his dog, had not occurred. However, it is possible that the dogs may have been colonized by more than one strain, but due to the limitations of our typing methodology, multiple strain colonization could not be determined. Polyclonal *S. aureus* colonization in humans has been shown, caused by exposure to diverse strains from exogenous sources, such as other individuals or pets [23]. Therefore, in cases of suspected zoonotic transmission, multiple isolates should be selected for testing to limit missing potential cases of multiple strain colonizations. Alternatively, it is also possible that the patient could have been infected from another unknown source.

All isolates in this case report were positive for the genes encoding toxins LukI and SIET, which is consistent with other reports on the detection of virulence genes in *S. pseudintermedius* [15]. These genes share significant homology with PVL and other exfoliatins detected in *S. aureus*. While *S. intermedius* exfoliative toxin has been shown to cause symptoms similar to canine pyoderma and human scalded skin syndrome, and LukI to be leucotoxic to polymorphonuclear cells, their role in pathogenesis among strains of *S. pseudintermedius* remains unclear [4]. While zoonotic transmission between the patient and his dogs could not be confirmed, to our knowledge, this is the first report of a *S. pseudintermedius* infection in a human from Scotland.
